# An unusual presentation of ischemic stroke

**DOI:** 10.11604/pamj.2020.36.172.24075

**Published:** 2020-07-10

**Authors:** Aziz Ahizoune, Ahmed Bourazza

**Affiliations:** 1 Department of Neurology and Neurophysiology, Mohammed V Military Teaching Hospital, University of King Mohammed V-Souissi, Rabat, Morocco

**Keywords:** Ataxia, ophtalmoplegia, stroke

## Images in medicine

*A 67-year-old man with history of diabetes mellitus and arterial hypertension, presented with sudden diplopia and gait disturbance. Physical examination showed truncal instability and dysmetria of left limbs, associated with signs of left unilateral internuclear ophtalmoplegia (INO), without impairment of convergence (A, B). Two days later, Brain MRI showed a small ischemic stroke involving the paramedian tegmentum of the left ponto-mesencephalic junction (C, D). The stroke was secondary to the atherosclerosis process. The patient was treated with 160mg/j of aspirin. The follow up at 10 days was marked with little attenuation of neurological signs*.

**Figure 1:**
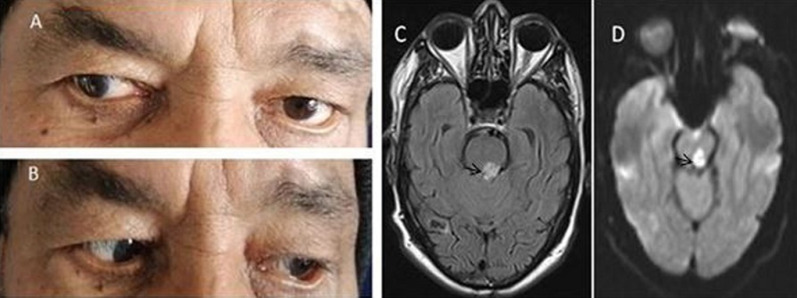
defective adduction of the left eye and nystagmus of the contralateral eye on abduction (A); normal abduction of the right eye and normal adduction of the contralateral eye (B); lesion showing hyperintensity in axial fluid-attenuated inversion recovery sequence of the left ponto-mesencephalic junction (C); diffusion restriction in the axial diffusion-weighted imaging sequence of the same lesion (D)

